# Factors influencing early response of IgA nephropathy following targeted-release budesonide (TRB) treatment: preliminary results from a multicenter study

**DOI:** 10.1093/ckj/sfae364

**Published:** 2024-11-19

**Authors:** Christodoulos Keskinis, Eleni Moysidou, Eleni Kapsia, Vasilios Vaios, Christos Bintas, Maria Trivyza, Michalis Christodoulou, Georgios Lioulios, Stamatia Stai, Christina Nikolaidou, Panagiotis Pateinakis, Marios Papasotiriou, Vassilios Liakopoulos, Smaragdi Marinaki, Maria Stangou

**Affiliations:** School of Medicine, Aristotle University of Thessaloniki (AUTH), Thessaloniki, Greece; Department of Nephrology, Papageorgiou Hospital, Thessaloniki, Greece; School of Medicine, Aristotle University of Thessaloniki (AUTH), Thessaloniki, Greece; 1^st^ Department of Nephrology AUTH, Hippokration Hospital, Thessaloniki, Greece; Department of Nephrology and Renal Transplantation, Medical School, National and Kapodistrian University of Athens, Laiko Hospital, Athens, Greece; 2^nd^ Department of Nephrology, Medical School, Aristotle University of Thessaloniki, AHEPA Hospital, Thessaloniki, Greece; Department of Nephrology and Renal Transplantation, Medical School, National and Kapodistrian University of Athens, Laiko Hospital, Athens, Greece; Department of Nephrology and Renal Transplantation, University Hospital of Patras, Patras, Greece; School of Medicine, Aristotle University of Thessaloniki (AUTH), Thessaloniki, Greece; 1st Department of Nephrology AUTH, Hippokration Hospital, Thessaloniki, Greece; School of Medicine, Aristotle University of Thessaloniki (AUTH), Thessaloniki, Greece; 1st Department of Nephrology AUTH, Hippokration Hospital, Thessaloniki, Greece; School of Medicine, Aristotle University of Thessaloniki (AUTH), Thessaloniki, Greece; 1st Department of Nephrology AUTH, Hippokration Hospital, Thessaloniki, Greece; Department of Pathology, Hippokration Hospital, Thessaloniki, Greece; Department of Nephrology, Papageorgiou Hospital, Thessaloniki, Greece; Department of Nephrology and Renal Transplantation, University Hospital of Patras, Patras, Greece; 2^nd^ Department of Nephrology, Medical School, Aristotle University of Thessaloniki, AHEPA Hospital, Thessaloniki, Greece; Department of Nephrology and Renal Transplantation, Medical School, National and Kapodistrian University of Athens, Laiko Hospital, Athens, Greece; School of Medicine, Aristotle University of Thessaloniki (AUTH), Thessaloniki, Greece; 1st Department of Nephrology AUTH, Hippokration Hospital, Thessaloniki, Greece

**Keywords:** histological findings, IgA nephropathy, proteinuria, targeted released budesonide treatment

## Abstract

**Background:**

Formation of galactose-deficient IgA1 (Gd-IgA1) immunoglobulin is the initial step in the immunological cascade leading to IgA nephropathy (IgAN). Targeted-release budesonide (TRB), an evidence-based regimen without major side-effects, has recently been approved for IgAN treatment; herein we present our preliminary real-world data regarding prompt response to TRB.

**Methods:**

Patients with primary IgAN who remained with Uprot >1 g/24 h despite conventional treatment for 6 months were started on TRB, and re-evaluated at 3 (T3) and 6 (T6) months. Reduction of proteinuria by ≥30%, at T3 and T6 was regarded as very early (VER) and early response (ER), respectively. Kidney biopsies were evaluated according to Oxford classification (MEST-C) score.

**Results:**

Thirty-seven IgAN patients, male/female 26/11, mean ± standard deviation age 50.38 ± 14.32 years and mean time since diagnosis 45.65 ± 56.67 months, were included. Seventeen (45.94%) patients demonstrated VER, increasing to 29 (78.3%) as ER (*P* = .004). Patients who demonstrated VER had a shorter time interval since diagnosis compared with non-VER, 29.41 ± 6.96 vs 65.37 ± 17.64 months (*P* = .05), and preserved estimated glomerular filtration rate at diagnosis and T0, while time since diagnosis was the main factor associated with ER, 38.36 ± 19.6 vs 78.67 ± 18.64 months, in ER and non-ER respectively (*P* = .05). Patients with M0, E0, S0 and T0 had no significant changes during T0–T6, while patients with M1, E1, S1 and even T1 had significantly reduced proteinuria (*P* = .006, *P* = .0011, *P* < .0001 and *P* < .0001, respectively).

**Conclusions:**

Almost half of the patients showed proteinuria reduction after TRB treatment at 3 months, and the proportion increased significantly at 6 months. Patients likely to have a prompt proteinuria reduction were relatively close to diagnosis, retained kidney function and had active lesions in kidney biopsy.

## Graphical Abstract

**Graphical Abstract:**
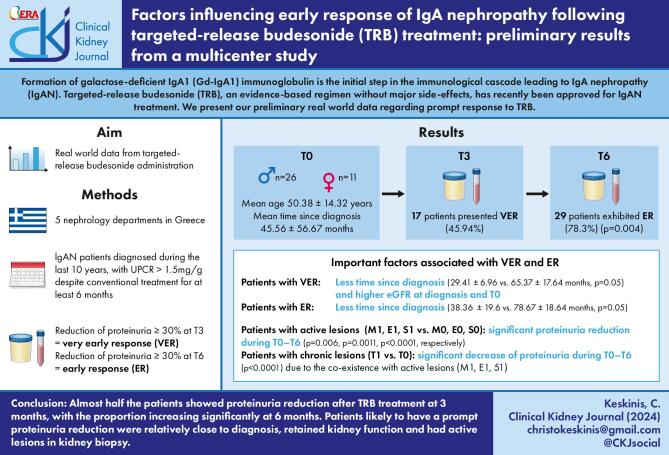

KEY LEARNING POINTS **What was known:**

- IgA nephropathy (IgAN) is the most common primary glomerulonephritis worldwide and has a relatively poor prognosis, as approximately 20% of patients will eventually reach end-stage kidney disease within 20 years of diagnosis.
- Conventional treatment should be applied in all patients, yet its effect on the local and systemic immune pathways is negligible. Steroids can have a beneficial effect in proteinuria, although their frequent side-effects and increased rate of relapses limit a broad use.
- Targeted-release budesonide (TRB) is the first treatment approach that aims to disease pathogenesis, and recent clinical studies have shown beneficial effect on proteinuria reduction and kidney function preservation.

**This study adds:**

- Our real-world clinical data following TRB treatment in IgAN patients showed that almost half of the patients experienced a very early reduction of proteinuria, at 3 months after treatment initiation, while the rate increased significantly at 6 months.
- Time since initial diagnosis and preserved kidney function were the main clinical parameters determined early response.
- Patients with active histological findings, including the presence of mesangial or/and endocapillary hypercellularity or/and segmental sclerosis could benefit from TRB treatment. Patients presenting T1 lesions had also their proteinuria levels significantly decreased compared with those with T0 lesions due to the co-existence with active lesions.

**Potential impact:**

- Evaluation of clinical parameters and histological findings seems to be important in order to choose and apply the best treatment approach for IgAN patients.
- TRB treatment seems to have great potential to achieve a prompt reduction of proteinuria, however it needs to be given early after diagnosis, preferably in patients with active lesions and preserved kidney function.

## INTRODUCTION

IgA nephropathy (IgAN), the most common primary glomerulonephritis worldwide, is typically manifested by a variable severity of hematuria, proteinuria and impaired kidney function, and usually moves slowly and inexorably towards chronic kidney disease [1, 2]. IgAN is undoubtedly a disease of immune origin, while the widely accepted theory of the “four-hit pathogenic” model is probably associated with the mucosal immune system. B lymphocytes in the mucosa-associated lymphoid tissue are conscripted to protect against pathogens, through the production of IgA immunoglobulin. In patients with IgAN, galactose-deficient IgA1 (Gd-IgA1) molecules formed by B lymphocytes circulate in the peripheral blood (Hit 1), stimulating secretion of IgA and/or IgG autoantibodies (Hit 2) and forming immune complexes (Hit 3) which finally deposit themselves in the mesangium, initiating tissue damage (Hit 4) [1, 3, 4]. Histological findings in IgAN consist of different and multiple lesions, covering a great spectrum of severity, ranging from mild mesangial hypercellularity to severe endocapillary and extra-capillary proliferation, sclerotic lesions and tubulointerstitial fibrosis, which have been classified and graded according to MEST-C classification score [5, 6].

In recent years, after a long journey, treatment of the disease has focused on glomerular inflammation and chronic kidney damage [1, 3, 4]. The most recently published KDIGO (2021) guidelines, apart from non-immunological modalities directed at preventing chronic kidney damage, highlight the circumstances under which immunological therapies may be administered for a short period of time [7]. In particular, systemic corticosteroids are considered an effective treatment over time. However, the occurrence of serious adverse events reported in recent trials have prompted the nephrology community to define certain circumstances under which they can be administered. Specifically, KDIGO 2021 guidelines suggest that the risk of treatment-related toxicity must be discussed with the patients, especially those with glomerular filtration rate (GFR) <50 mL/min/1.73 m^2^. The same recommendation, graded as 2B, suggests that patients who fulfill certain criteria and are at high risk for disease progression, despite maximal non-immunological treatment, can be considered for a 6-month course of systemic corticosteroid administration [1, 2]. The clinical benefit of glucocorticoids administration must be assessed with caution in the following situations: diabetes, obesity, GFR <30 mL/min/1.73 m^2^, cirrhosis, severe osteoporosis, peptic ulcer and latent infections [7]. However, recent accumulating evidence suggests that targeted treatment should aim to tackle the cause of the disease and intervene at early stages of disease pathogenesis, and not merely aim to restore glomeruli damage [1, 8]. Furthermore, IgAN is a chronic disease, characterized by sustained and low-grade inflammation, and frequently relapse after steroid or immunosuppressive treatment, yet the chronicity of the disease acts as a deterring factor for long-term immunosuppressive treatment.

We conducted the present study based on recent findings that introduced targeted-release budesonide (TRB) as a novel treatment for IgAN, in order to evaluate its effect in a real-world clinical practice, estimate its early effects, and assess clinical and histologic parameters that potentially facilitate response.

## MATERIALS AND METHODS

In the present prospective, open label, multi-center study, patients with IgAN, diagnosed during the last 10 years, who fulfilled the inclusion criteria described below were initiated on TRB treatment, 16 mg daily for 9 months, followed by 1-month tapering. The patients were followed on a regular basise of 3-month intervals. The whole study was designed based on the regular treatment approach, and follow-up was scheduled according to the common practice for IgAN. Therefore, the study was not performed under the strict controlled conditions of a randomized control trial, but rather as common practice, presenting real-world data (RWD) and aiming to assess the potential benefits of TRB in the everyday clinical practice, and the importance of histology and co-existing parameters to disease outcome. The Chronic Kidney Disease Epidemiology Collaboration (CKD-EPI) equation was applied to estimate GFR (eGFR), and severity of proteinuria was based on 24-h protein levels (Uprot) and urine protein/creatinine ratio (UPCR). The above parameters were recorded at the time of diagnosis (Tbx), at the beginning of the study (T0), and 3 months (T3) and 6 months (T6) after commencing treatment. Our study's inclusion and exclusion criteria are presented below:

Inclusion criteria:

- Age ranging from 18 to 75 years
- Caucasian race
- Primary IgAN confirmed by kidney biopsy
- The time period since the diagnosis not more than 10 years
- eGFR >30 mL/min/1.73 m^2^ according to CKD-EPI equation
- Uprot >1 g/day in two consecutive measurements at least 1 month apart
- Treatment with maximal tolerated dose of renin–angiotensin–aldosterone axis inhibitors (RAASi) and/or sodium-glucose co-transporter 2 inhibitor for at least 6 months before joining the study
- Written consent

Exclusion criteria:

- IgA vasculitis or secondary IgAN
- Presence of diabetes mellitus (pre-diabetic patients, adequately controlled, achieving HbA1C levels 5.5%–6.4%, only with diet, and no need for antidiabetic treatment, were included)
- Liver cirrhosis
- Treatment with steroids or immunosuppressives during the last 3 months; it is noted that all participants had not received immunosuppressives for at least 6 months before joining the study

### Definitions

Reduction of proteinuria by ≥30%, combined with stable eGFR at T3 and T6, was regarded as very early response (VER) and early response (ER), respectively.

Infections during treatment were characterized as non-severe if they were not life-threatening and no hospitalization was not required.

Increased blood pressure (BP) was defined as systolic BP ≥140 mmHg and/or diastolic BP ≥90 mmHg.

### Schedule of the study

The Bioethics and Ethics Committee of the Faculty of Medicine of the Aristotle University of Thessaloniki has approved the study, ID Number 209/2024.

Kidney biopsies were re-evaluated independently by one pathologist and one nephrologist, who were unaware of clinical data, and classified according to the 2016 revised Oxford classification system [9]. In case of interobserver disagreement, biopsies were evaluated and classified by a nephropathologist.

Evaluation and monitoring were scheduled as follows:

- At time point T0: history and clinical findings • Time elapsed since the diagnosis of IgAN • Record of previous immunosuppressive treatment
- At time point T0: histological pattern of kidney biopsy • Scoring kidney biopsies based on the Oxford classification system (MEST-C score)
- At time points T0, T3, T6: laboratory findings • eGFR CKD-EPI, Uprot and UPCR were evaluated

Based on MEST-C score, patients were classified as M0 and M1, E0 and E1, S0 and S1, based on the absence or presence of mesangial hypercellularity, endocapillary hypercellularity and focal sclerosis, respectively, T0, T1 and T2, with tubular atrophy in 0%–25%, in 26%–50% and >50% of tubules, respectively, and C0 and C1, with no or crescents in one or more glomeruli, respectively.

Primary endpoints were the presence of VER and ER and the possible impact of histology, as defined by Oxford classification. Secondary endpoints were the outcome of eGFR CKD-EPI, Uprot and UPCR at T3 and T6 time points, respectively, after 6 months of TRB treatment and determination of other prognostic factors which may affect treatment's effectiveness.

### Statistics

IBM SPSS 26.0 (SPSS Inc., Chicago, IL, USA) program was used for statistics, *P*-values <.05 were considered statistically significant and normality of all quantitative variables was estimated by Shapiro–Wilk test, and accordingly, mean and standard deviation or median value and interquartile range were used for description of the results, while T-test for independent and dependent variables, and Mann–Whitney U test and Wilcoxon test were applied to compare the mean or median values of two independent or dependent groups, respectively. Similarly, analysis of variance (ANOVA) test and Kruskal–Wallis H test were used to estimate differences between more than two groups, respectively.

Multiple regression analysis was performed to define the independent factors that contributed to proteinuria levels, as the dependent variable, at T3 and T6. The regression models created included the most important histological, combined with some clinical parameters, such as renal function. The R^2^ levels determined the percentage of the dependent variable (proteinuria) explained by the independent ones, beta coefficients were estimated to express the expected change in the dependent variable whereas partial coefficients were estimated to express the same change, assuming that all other predictor variables are held constant.

All grafts were performed using the GrafPad Prism9 program.

## RESULTS

**Figure 1:**
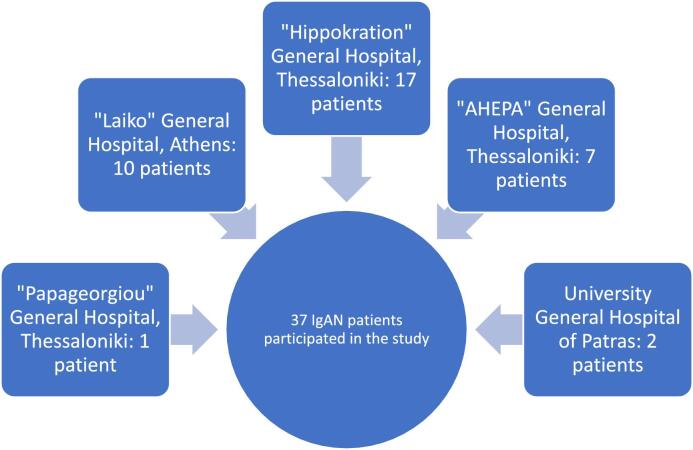
Recruitment of patients from each participating center.

Patients who participated in the study are still under treatment and their monitoring is under evaluation. However, here we present the preliminary results from 37 patients who have already completed 6 months of treatment (Fig. 1).

The majority of patients were males (26/37), with a mean ± standard deviation age of 50.38 ± 14.26 years, while mean time since diagnosis was 45.65 ± 56.67 months. Seventeen patients (45.94%) had received systemic corticosteroids and two (5.4%) cyclophosphamide in the past. Time since systemic corticosteroid administration was 12.53 ± 2.81 months, 16/17 had finished steroid treatment at least 8 months prior to their enrolment in the study, only 1/17 had completed steroid treatment 6 months before he starting on TRB. Cyclophosphamide was administrated to only two patients before 11 and 15 months, respectively. Meanwhile, all patients were treated with RAASi, and 23 (62.16%) with sodium-glucose co-transporter 2 inhibitors (SGLT2i), for at least 6 months, before starting TRB treatment [10]. Clinical and epidemiologic data at T0, for all 37 patients participated in the study are presented in Table 1.

**Table 1:** Clinical and epidemiologic data of the 37 patients participated in the study and received TRB, at time of starting treatment (T0).

Clinical parameters at induction (T0)	All patients
*N*	37
M/F	26/11
Age (years)	50.38 ± 14.26
Systolic BP (mmHg)	129.38 ± 9.47
Diastolic blood pressure (mmHg)	75 ± 11.55
Smoking habits, *n* (%)	26 (70.27)
BMI (kg/m^2^)	25.68 ± 3.45
Statin administration, *n* (%)	26 (70.27)
Time since diagnosis (months)	45.65 ± 56.67
Previous steroid treatment, *n* (%)	17(45.94)
Previous cyclophosphamide treatment, *n* (%)	2 (5.4)
Current RAASi treatment, *n* (%)	37 (100)
Current SGLT2i treatment, *n* (%)	23 (62.16)
eGFR (mL/min/1.73 m^2^)	57.29 ± 23.52
Uprot (g/24 h)	2.83 ± 1.6

Data are presented as mean ± standard deviation or *n* (%).

### RAASi—BP control

BP measurements and concomitant dosage of RAASi (37/37) and SGLT2i (23/37) were recorded at time points T0, T3 and T6 during treatment. No statistically significant changes were detected among BP measurements. All 37 patients had already been treated for at least 6 months with RAASi and 23 of them were also on SGLT2i for the same time period. Anti-proteinuric treatment was decided by> the treatment physician, and no changes were applied after entering the study, or during the first 6 months of TRB treatment. Nineteen patients (51.35%) were on ramipril 5 mg/day, 10/37 (27.02%) irbesartan 150–300 mg/day, 4/37 (10.81%) lisinopril 10 mg/day and 4/37 (11.11%) patients were treated with candesartan, of whom 3 patients received 32 mg/day and one patient received 16 mg/day. During the study period, apart from a temporary increase in BP noticed in one case, which was treated with a reduction in TRB dose, no statistical difference was detected among the measurements and no adjustments in RAAS and SGLT2 inhibition regimens were necessary. Table 2 describes type and dosage of RAASi and SGLT2i and mean values of the BP measurements at each timepoint during treatment.

**Table 2:** Overview of non-immunological antiproteinuric treatment and mean values of participants’ BP measurements during the treatment with TRB.

			Timepoints of treatment					
			T0		T3		T6	
			SBP (mmHg)	DBP (mmHg)	SBP (mmHg)	DBP (mmHg)	SBP (mmHg)	DBP (mmHg)
RAASi, *n* (%)		SGLT2i, *n*	129.38 ± 9.47	75 ± 11.55	126.88 ± 7.72	77.14 ± 8.02	127.5 ± 8.03	76.07 ± 9.24
Ramipril	19 (51.35%)	12	5 mg/day					
Irbersartan	7 (18.92%)	4	150 mg					
	3 (8.1%)	2	300 mg					
Lisinopril	4 (10.81%)	3	10 mg					
Candesartan	3 (8.1%)	2	32 mg					
	1 (2.7%)		16 mg					
Total	37	23 (62.16%)						

DBP, diastolic BP; SBP, systolic BP.

### Disease outcome before and after commencing TRB treatment

During the time interval between biopsy (Tbx) and start of TRB treatment (T0) (45.69 ± 56.67 months), eGFR was changed in our 37 participants, from 61 ± 23.42 (Tbx) to 57.29 ± 23.52 mL/min/1.73 m^2^ (T0) (*P* = .09), and Uprot from 2.81 ± 1.88 g/24 h (Tbx) to 2.83 ± 1.6 g/24 h (T0) (*P* = .78).

**Figure 2:**
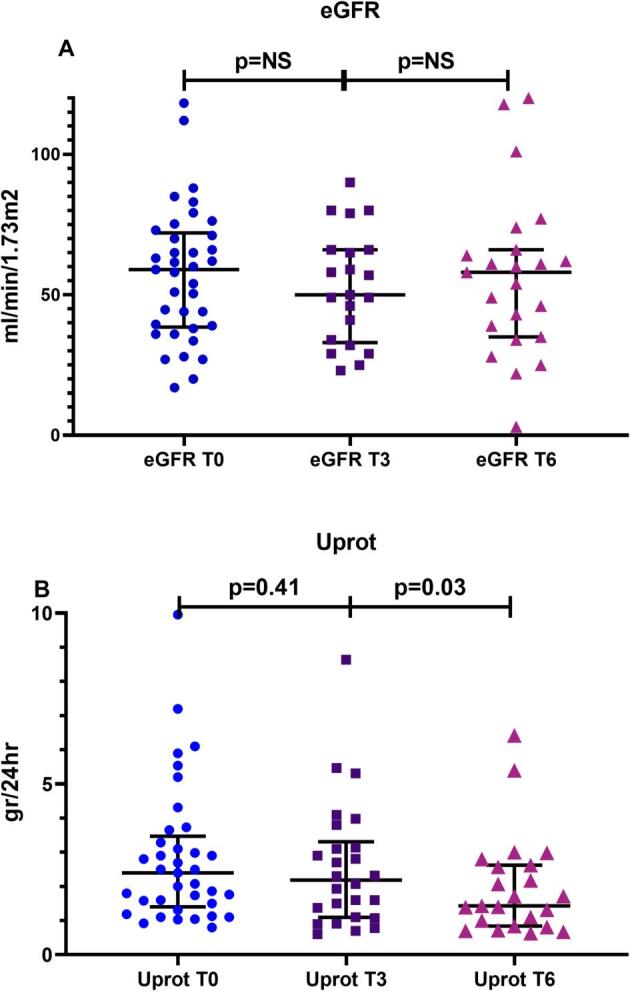
Outcome of renal function (eGFR) (**A**) and proteinuria (Uprot) (**B**) after starting on TRB treatment, in the whole cohort of patients, at T0, T3 and T6.

After starting TRB treatment, in the whole cohort of patients, kidney function remained stable, eGFR was 57.29 ± 23.52, 52.71 ± 19.91 and 58.90 ± 26.3 mL/min/1.73 m^2^ (*P* = .78) at the time points T0, T3 and T6, respectively, while proteinuria was gradually reduced, from 2.83 ± 1.6 (T0) to 2.56 ± 1.85 g (T3) and 1.98 ± 1.47 g (T6), respectively (*P* = .009) (Fig. 2).

### VER after starting TRB treatment

Seventeen patients (45.94%) had a VER, as this was defined by the combination of reduction in Uprot by ≥30% and stable eGFR, after 3 months of TRB treatment.

Changes in eGFR and proteinuria levels in the 17 patients with VER, from T0 to T3 are described in Table 3.

**Table 3:** Changes in kidney function and proteinuria in VER and non-VER patients (from T0 to T3) and in ER and non-ER patients (from T0 to T3 and T6).

VER			Non-VER			ER			Non-ER		
*n* = 17			*n* = 20			*n* = 29			*n* = 8		
eGFR (mL/min/1.73 m^2^)			eGFR (mL/min/1.73 m^2^)			eGFR (mL/min/1.73 m^2^)			eGFR (mL/min/1.73 m^2^)		
T0	T3	*P*	T0	T3	*P*	T0	T3	*P*	T0	T3	*P*
61.11 ± 16.91	60.77 ± 18.79	.28	44.21 ± 17.7	46.66 ± 19.26	.22	53.07 ± 19.89	54.07 ± 19.07	.61	51.2 ± 16	53.4 ± 23.71	.7
						**T3**	**T6**	***P***	**T3**	**T6**	***P***
						54.07 ± 19.07	52.21 ± 18.34	.61	53.4 ± 23.71	46.4 ± 18.39	.16
**Uprot (g/24 h)**			**Uprot (g/24 h)**			**Uprot (g/24 h)**			**Uprot (g/24 h)**		
**T0**	**T3**	***P***	**T0**	**T3**	***P***	**T0**	**T3**	***P***	**T0**	**T3**	***P***
2.67 ± 1.61	1.47 ± 0.81	.002	2.96 ± 1.69	3.49 ± 1.98	.9	3.06 ± 1.79	2.3 ± 1.51	.014	2.38 ± 1	3.71 ± 2.97	.26
						**T3**	**T6**	***P***	**T3**	**T6**	***P***
						2.3 ± 1.51	1.58 ± 0.82	.03	3.71 ± 2.97	3.38 ± 2.4	.62
**UPCR (g/g)**			**UPCR (g/g)**			**UPCR**			**UPCR**		
**T0**	**T3**	***P***	**T0**	**T3**	***P***	**T0**	**T3**	***P***	**T0**	**T3**	***P***
2.26 ± 0.54	1.37 ± 0.45	.1	1.89 ± 0.2	2.15 ± 0.35	.61	1.9 ± 0.64	1.94 ± 1	.37	2.15 ± 0.9	2.2 ± 1.2	.14
						**T3**	**T6**	***P***	**T3**	**T6**	***P***
						1.94 ± 1	1.73 ± 0.89	.89	2.2 ± 1.2	2.74 ± 1.24	.12

Data are presented as mean ± standard deviation.

### Parameters associated with VER

As described in Table 4, patients with VER had been relatively recently diagnosed with IgAN, and were slightly younger than non-VER patients. They also had a significantly preserved renal function and higher levels of proteinuria at Tbx and at T0, compared with non-VER patients. Proteinuria and UPCR levels did not have any significant difference between VER and non-VER patients at T0. Moreover, many other parameters were also evaluated. In detail, systolic and diastolic BP, smoking habits, body mass index (BMI) and the statin administration were also evaluated, but no statistically significant modification was detected.

**Table 4:** Differences in clinical and laboratory findings between VER and non-VER patients after 3 months of TRB treatment.

	VER	Non-VER	*P*
*N*	17	20	
Male/female	13/4	13/7	.45
Age (years)	45.5 ± 13.73	52.36 ± 12.95	.2
SBP (mmHg) T3	128.33 ± 10.41	126.5 ± 8.18	.84
DBP (mmHg) T3	76.67 ± 5.77	76 ± 10.49	.98
Smoking habits (*n*)	11	15	.97
BMI (kg/m^2^)	25.32 ± 2.98	26.00 ± 3.86	.55
Statin administration (*n*)	8	18	.23
Time since diagnosis (months)	29.41 ± 6.96	65.37 ± 17.64	.05
eGFR Tbx (mL/min/1.73 m^2^)	70.6 ± 24.74	52.46 ± 16.35	.05
eGFR T0 (mL/min/1.73 m^2^)	67.96 ± 21.98	48.05 ± 19.24	.01
Uprot Tbx (g/24 h)	3.64 ± 2.37	2.15 ± 1.24	.05
Uprot T0 (g/24 h)	2.67 ± 1.61	2.96 ± 1.69	.66
UPCR Tbx (g/g)	3.86 ± 0.68	1.83 ± 1.14	.02
UPCR T0 (g/g)	2.26 ± 0.54	1.89 ± 0.2	.66

Data are presented as mean ± standard deviation or *n*.

SBP, systolic BP; DBP, diastolic BP.

**Figure 3:**
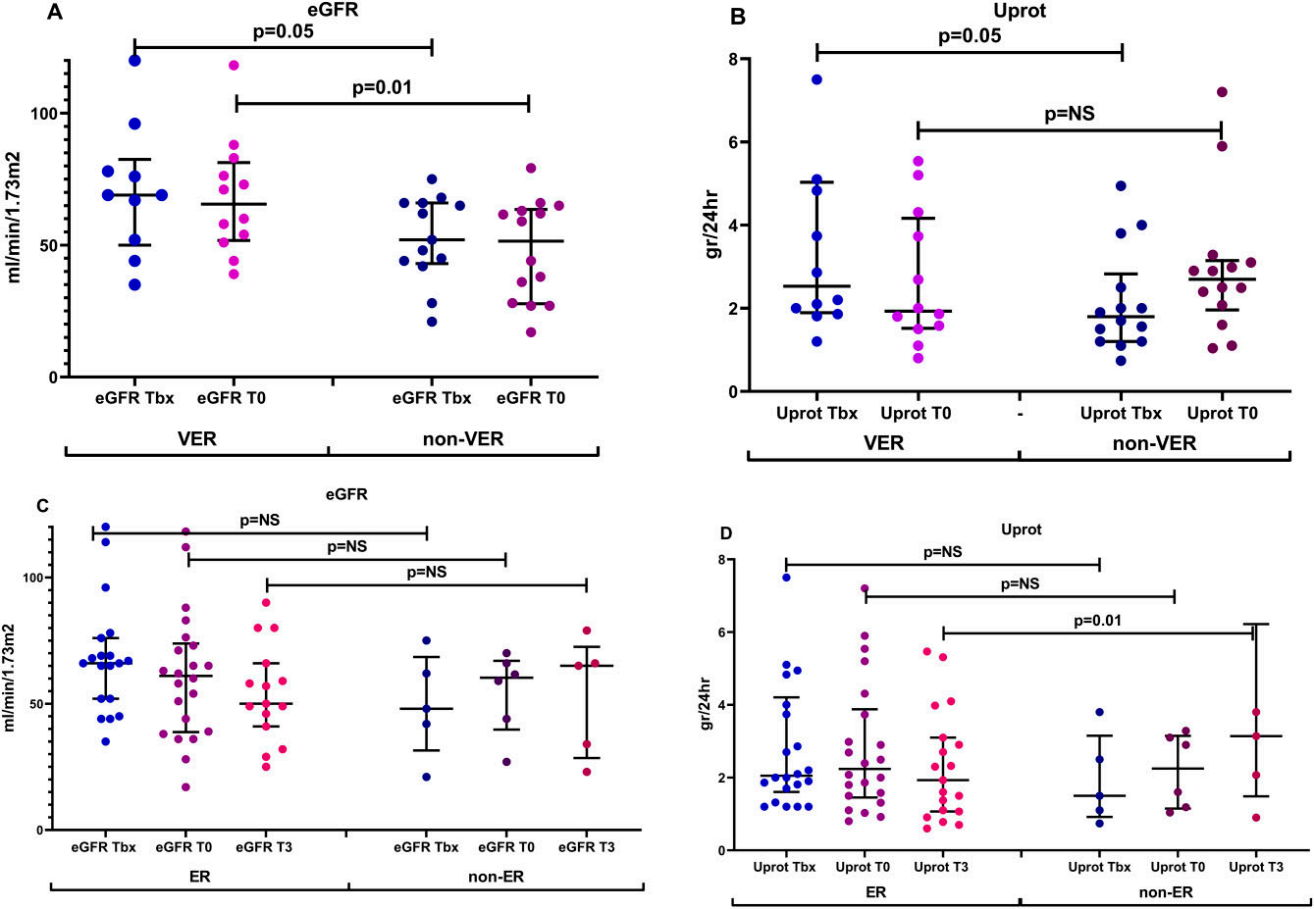
Differences between eGFR and Uprot levels at Tbx and T0 in VER (**A**) and non-VER (**B**) patients, and eGFR and Uprot levels at Tbx, T0 and T3 in ER (**C**) and non-ER (**D**) patients.

Differences in eGFR and Uprot between VER and non-VER patients at Tbx and T0 are depicted in Fig. 3.

### ER after starting TRB treatment

At the end of 6 months of treatment, the prevalence of proteinuria reduction significantly increased, therefore, the 17/37 patients with VER presented also ER, and 12 more patients were added, increasing the number with ER to 29/37 (78.3%) (*P* = .004). The evolution of eGFR and Uprot levels in those with and without ER is demonstrated at Table 3.

### Parameters associated with ER

Patients with ER had better renal function at Tbx and T0, however differences were not statistically significant. The main differences between ER and non-ER patients was the degree of Uprot at T3 2.07 ± 1.02 vs 3.5 ± 1.89 g/24 h (*P* = .01). Moreover, time elapsed since diagnosis (38.36 ± 19.6 vs 78.67 ± 18.64 months; *P* = .05) was also an important factor, but not statistically significant (Table 5, Fig. 3).

**Table 5:** Differences in clinical and laboratory findings between ER and non-ER patients.

	ER	Non-ER	*P*
*N*	29	8	
Male/female (*n*)	20/9 (pts)	6/2	
Age (years)	47.68 ± 14	52.83 ± 12.95	.42
SBP T6 (mmHg)	126.82 ± 7.33	130 ± 10	.56
DBP T6 (mmHg)	75 ± 9.49	80 ± 8.67	.42
Smoking habits (*n*)	10	1	.24
BMI (kg/m^2^)	25.55 ± 3.21	26.2 ± 4.44	.64
Statin administration (*n*)	19	7	.24
Time since diagnosis (months)	38.36 ± 19.6	78.67 ± 18.64	.05
eGFR Tbx (mL/min/1.73 m^2^)	67.9 ± 22.47	49.6 ± 20.47	.11
eGFR T0 (mL/min/1.73 m^2^)	60.8 ± 25.2	54.6 ± 16.18	.57
eGFR T3 (mL/min/1.73 m^2^)	54.06 ± 19.06	53.4 ± 23.7	.95
Uprot Tbx (g/24 h)	2.99 ± 2.05	1.9 ± 1.2	.27
Uprot T0 (g/24 h)	2.78 ± 1.79	2.18 ± 1.02	.44
Uprot T3 (g/24 h)	2.07 ± 1.02	3.5 ± 1.89	.01
UPCR Tbx (g/g)	2.39 ± 1.44	1.28 ± 0.5	.48
UPCR T0 (g/g)	1.9 ± 0.35	2.15 ± 0.23	.56
UPCR T3 (g/g)	2.07 ± 1.02	3.53 ± 1.89	.12

Data are presented as mean ± standard deviation or *n*.

SBP, systolic BP; DBP, diastolic BP.

### Outcome of the disease in patients receiving SGLT2i

The same analysis was performed in the 23 patients receiving SGLT2i together with TRB. Twelve of them (51.17%) presented VER and 19/23 (82.6%) ER (*P* = .44). Co-administration of TRB with SGLT2i showed statistically significant increase of eGFR after 3 months. On the other hand, only 5/14 (35.71%) patients who did not receive SGLT2i presented VER, and 10/14 (71.42%) ER. All these findings are summarized in Table 6.

**Table 6:** Monitoring the kidney function and the treatment response of patients receiving both TRB and SGLT2i.

	TRB + RAASi + SGLT2i				TRB + RAASi			
*N*	23				14			
Time points	T0	T3	T6	*P*	T0	T3	T6	*P*
eGFR (mL/min/1.73 m^2^)	56.38 ± 17.44	56.08 ± 19.98	57.8 ± 19.7	.61	57.32 ± 30.99	46.12 ± 20.36	56.07 ± 36.48	.41
Uprot/24 h (g)	2.57 ± 1.54	2.14 ± 1.45	1.59 ± 1.47	.045	3.13 ± 2.48	2.58 ± 1.33	1.92 ± 0.78	.082
UPCR (g/g)	2.43 ± 0.42	2.4 ± 1.2	1.79 ± 0.77	.84	1.58 ± 0.46	1.89 ± 0.85	1.68 ± 0.89	.6
VER		12				5		.32
ER			19				10	.42

Data are presented as mean ± standard deviation or *n*.

### The impact of kidney biopsy findings in reduction of proteinuria

All kidney biopsies from included patients were classified according to MEST-C Oxford classification score. Each patient's MEST-C score is depicted in Supplementary data, Table S1.

**Figure 4:**
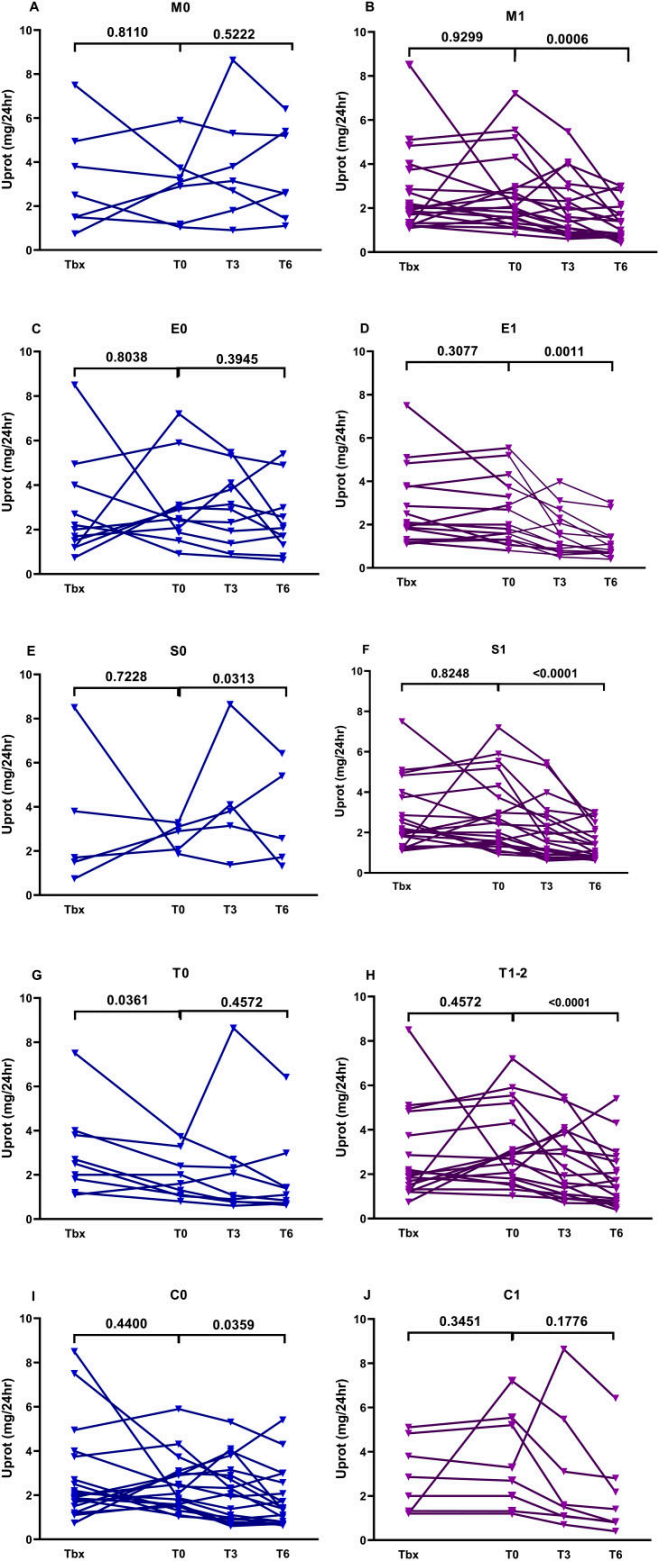
Changes in Uprot from Tbx to T0 and during the 6 months of TRB treatment, different outcome in patients with M0, E0, S0, T0, C0 (**A, C, E, G, I**, respectively) and M1, E1, S1, T1, C1 (**B, D, F, H, J**, respectively). *P*-values express significance in Uprot changes between the time interval Tbx-T0 (T-test) and changes between T0-T3-T6 (ANOVA test).

Patients who were classified as M1, E1 and S1 showed significant improvement in renal function and proteinuria after starting TRB treatment, compared with M0, E0 and S0 patients, respectively (Fig. 4). The presence of tubular atrophy, classified as T0, T1 and T2 and the presence of crescents, C0 and C1, did not seem to have any significant impact in response to treatment.

In order to estimate the impact of different combinations of parameters in the Oxford classification system, we assumed that M and E usually represent active and S and T chronic lesions, and we further sub-divided patients according to their MEST score, as follows:

- M1E0S0T0 or M0E1S0T0 corresponded to score 0 (mild activity, no chronicity)
- M0E0S1T0 or M0E0S0T1 corresponded to score 1 (no activity, mild chronicity)
- M1E0 or M0E1 or M1E1 and S1T0 or S0T1 corresponded to score 2; this score was also divided into 2a including patients with M1E0S1T0 or M1E0S0T1 (mild activity, mild chronicity) and into 2b including M1E1S1T0 or M1E1S0T1 (severe activity, mild chronicity)
- M1E0S1T1 or M0E1S1T1 and corresponded to score 3 M1E1S0T1 (mild activity, severe chronicity) and
- M1E1S1T1 corresponds to score 4 M1E1S0T1 (severe activity, severe chronicity)

Table 7 describes the correlation among sub-classification groups and VER and ER.

**Table 7:** Sub-classification of patients according to the overall histological findings of each biopsy.

Sub-classification		Number of patients		VER	Non-VER	ER	Non-ER
0 (mild activity, no chronicity)		2		0	2	1	1
1 (no activity, mild chronicity)		1		0	1	0	1
2	2a (mild activity, mild chronicity)	12	4	2	2	3	1
	2b (severe activity, mild chronicity)		8	6	2	7	1
3 (mild activity, severe chronicity)		8		5	3	8	0
4 (severe activity, severe chronicity)		14		10	4	10	4
Total		37		17	20	29	8

Two patients were classified as “0” score, they both had M0E1S0T0 score. None of them had VER and one of them had ER. Only one patient was classified as “1” score (M0E0S0T1) and he did not exhibit either VER or ER. Twelve patients were classified as “2” score. In particular, three patients who had M1E0S1T0 and one who had M1E0S0T1 score were graded as “2a” score. One patient with M1E0S1T0 and the only patient with M1E0S0T1 showed VER, while two patients with M1E0S1T0 and the patient with M1E0S0T1 showed ER. Eight participants had similar MEST score from their kidney biopsy (M1E1S1T0) and they were classified as “2b” score. Six of them (75%) had VER and seven of them (87.5%) showed ER. In addition, eight patients had M1E0S1T1 and they corresponded to “3” score. Five of them (62.5%) presented VER and all of them (100%) showed ER. Fourteen patients had M1E1S1T1 score, classified as “4,” and 10 of them (71.42%) showed VER and exactly the same patients presented ER.

Moreover, we took into consideration the amount of globally sclerosed glomeruli. Patients with VER had 4.7 ± 2.87 (23.65 ± 19.39%) sclerosed glomeruli versus 5.11 ± 7.19 (25.70 ± 13.84%) of patients without VER (*P* = .813). Meanwhile, patients with ER presented 5.78 ± 4.79 (27.08 ± 16.57%) sclerosed glomeruli, while non-ER exhibited 5.25 ± 2.60 (16.38 ± 13.52%) sclerosed glomeruli (*P* = 0.83).

### Multiple regression analysis

The most important parameters contributing to proteinuria levels, at the time points T3 and T6, were evaluated by creating different models, which included either histological findings based on Oxford classification system, or combined clinical indices and histological features.

Table 8 describes the models generated to predict proteinuria levels at T3 and T6, by multiple regression analysis. The presence of mesangial hypercellularity was the main independent factor contributing to proteinuria levels at both time points, while the severity of proteinuria at T0 and T3 also seemed to have significant impact in the T3 and T6 proteinuria levels, respectively (Table 9).

**Table 9:** Models created by multiple regression analysis to predict the proteinuria levels at time points T3 and T6.

Tested parameters	Enetered variables	*R* square	Adjusted *R* square	Std error	Stand coefficients B	*P*	CI	
Models predicting Uprot at T3								
M, E, S, T, C	M	0.451	0.421	1.44	–0.672	<.001	–6.39	–1.87
M, S, eGFR Tbx	M	0.451	0.415	1.55	–0.672	.003	–6.6	–1.6
M, S, Uprot Tbx	M	0.451	0.421	1.44	–0.672	.001	–6.39	–1.88
M, S, eGFR/Uprot Tbx	M	0.451	0.415	1.55	–0.672	.003	–6.6	–1.61
M, S, eGFR T0	M	0.451	0.421	1.44	–0.672	.001	–6.39	–1.87
M, S, Uprot T0	M	0.659	0.619	1.16	–0.639	.001	–5.77	–2.01
	Uptor T0				0.457		0.187	0.89
M, S, eGFR/Uprot T0	M	0.659	0.619	1.16	–0.639	<.001	–5.77	–2.01
	Uptor T0				0.457		0.187	0.89
Models predicting Uprot at T6								
M, E, S, T, C	M	0.756	0.739	0.841	–0.869	<.001	–5.96	–2.96
M, S, eGFR Tbx	M	0.754	0.736	0.869	–0.868	<.001	–5.71	–2.89
M, S, Uprot Tbx	M	0.756	0.739	0.841	–0.868	<.001	–5.71	–2.89
M, S, eGFR/Uprot Tbx	M	0.754	0.736	0.869	–0.868	<.001	–5.71	–2.89
M, S, eGFR T0	M	0.756	0.739	0.841	–0.869	<.001	–5.96	–2.96
M, S, Uprot T0	M	0.756	0.739	0.841	–0.869	<.001	–5.66	–2.96
M, S, eGFR/Uprot T0	M	0.756	0.739	0.841	–0.869	<.001	–5.66	–2.96
M, S, eGFR T3	M	0.771	0.752	0.855	–0.878	<.001	–5.57	–2.73
M, S, Uprot T3	M	0.875	0.855	0.629	–0.578	<.001	–4.19	–1.42
	Uptor T3				0.444		0.12	0.58
M, S, eGFR/Uprot T3	M	0.871	0.848	0.670	–0.599	<.001	–4.33	–1.32
	Uptor T3				0.422		0.08	0.59

### Adverse events

Some adverse events were observed during the first 6 months of treatment. Three patients (8.1%) experienced non-severe infection, one of them caused by SARS-CoV-2. One patient (2.7%) who had pre-diabetes before entering the study, showed an increase of glycated hemoglobin to over 8%, 3 months after starting TRB treatment and was commenced on antidiabetic treatment, 1 (2.7%) showed a temporal increase in BP and 1 (2.7%) female patient temporarily presented menstrual disorders. One patient had to discontinue treatment because she presented severe peripheral myopathy at the end of the fourth month of treatment; however, the patient remained on regular follow-up. All the adverse events are summarized in Table 8.

**Table 8:** Adverse effects presented during the first 6 months, in the 37 patients treated with TRB.

Side effects	Number of patients	Continue with reduced treatment dosage	Treatment discontinuation
Acne	3	0	0
Infection	3	0	0
Dysregulation of pre-diabetic status	1	0	0
High BP	1	1	0
Menstrual disorders	1	0	0
Myopathy	1	0	1

## DISCUSSION

The present study aimed to demonstrate prompt effects of TRB treatment and estimate clinical and histological parameters used in the everyday clinical practice that could assist in prediction to treatment response.

As the patients’ outcome was assessed at very short time intervals after initiating TRB treatment, we decided to estimate the trend in proteinuria reduction, as this was defined by ≥30% from baseline, and not the standard reduction of <1 g/24 h described in recent guidelines [7]. Based on this definition, treatment with TRB combined with other anti-proteinuric agents, demonstrated a very early reduction in proteinuria, in almost half of the patients at the end of 3 months, whilst this proportion was significantly increased at the end of 6 months of treatment.

Specific parameters can indeed predict response to IgAN treatment. They have been studied thoroughly and it is considered that hypertension, impaired renal function and increased levels of time averaged proteinuria are factors associated with worse outcome. Most of the studies however have been performed in patients receiving steroids and/or angiotensin-converting enzyme inhibitors [11–15]. TRB is a new treatment, which only recently obtained Food and Drug Administration and European Medicines Agency approval for IgAN, and data regarding factors to predict response to TRB are still under investigation [16].

Preserved kidney function at diagnosis and at the time point of TRB treatment initiation (T0), as well as the relatively short time interval since initial diagnosis, were important clinical parameters associated with a very early response, after 3 months of treatment. Most importantly, all patients achieving proteinuria reduction at 3 months continued to improve, suggesting that prompt reduction in proteinuria may be regarded as an indicator of more sustained response. Therefore, after 6 months TRB treatment, the proportion of patients with reduced proteinuria had risen to 78%, their main characteristic being the short time interval since diagnosis. Although the unfavorable impact of chronicity in the response to treatment is not surprising, the exact way this long-term existence of disease will affect outcome is not clear, and has not been thoroughly investigated. Long-term sustained and untreated disease is followed by progressive tubular atrophy and interstitial fibrosis, lesions that cannot be reinstated with any immunosuppressive or anti-inflammatory treatment [17, 18]. In the present study we demonstrate that patients with long-term disease and those with advanced tubulointerstitial and no active lesions had no adequate response to TRB, while patients with active histology suggestive of active inflammation, including mesangial and/or endocapillary hypercellularity and/or segmental sclerosis showed a significant reduction in proteinuria. This is a remarkable finding, as the impact of histology in response to TRB treatment has not been investigated before.

Mesangial (M) and endocapillary cellularity (E), segmental sclerosis (S), interstitial fibrosis/tubular atrophy (T) and the presence of crescents (C) were assessed to form the MEST-C score, initially described in 2009, modified in 2017 and widely accepted as the histological classification system in IgAN. In previous studies the presence of M, S and T proved to correlate with prognosis, T lesions reflected the stage of the disease at time of kidney biopsy and correlated with advanced kidney damage, while immunosuppressive treatment had significant impact in patients with endocapillary cellularity [17, 18]. We subdivided the patients, based on the combination of active and chronic histological lesions, and tried to classify our findings for a more precise evaluation. Particularly, 12 patients were classified as having mild or severe activity and mild chronicity (M1E0S1T0, M1E0S0T1 or M1E1S1T0), 8 patients presented with mild activity and severe chronicity (M1E0S1T1) and 4 patients had severe activity with severe chronicity (M1E1S1T1). Although the number of participants was small, it becomes obvious that patients with active lesions had a rapid reduction in proteinuria, regardless of the co-existence of chronic lesions. The aforementioned data suggest that the presence of tubular atrophy, which is a chronic lesion, combined with M1 or/and E1 cannot limit the potential benefit of TRB administration. Apparently, a long-term follow up is critical in order to estimate the particular predictive value of advanced histology.

The development of secondary glomerular sclerosis in IgAN, as a consequence of IgA1 immune deposition, stimulation of mesangial cells, subsequent release of cytokines and inflammatory mediators responsible for the mesangial–podocyte cross talk, which induce podocyte injury, maybe totally different from the development of focal segmental glomerulosclerosis in other primary glomerulonephritis such as membranous or membranoproliferative glomerulonephritis [19–23]. Moreover, the type of glomerulosclerosis, focal or global sclerosis, in IgAN seems to play critical role in the response to immunosuppressive treatment and in predicting renal function outcome [17].

Some investigators support that although mesangial and endothelial hypercellularity can be ameliorated by treatment, focal segmental sclerosis, as a chronic lesion, cannot be resorted [24]. We found that M, E and S were parameters that favored response to TRB treatment, although mesangial hypercellularity was the most important independent factor to predict severity of proteinuria.

Our findings indicate that patients with active disease, as demonstrated by histology and severity of proteinuria, preserved kidney function and relatively short period since disease initial diagnosis, are most likely to promptly reduce proteinuria even after 3–6 months of TRB treatment. The precise assessment of clinical and histological findings in order to choose the most appropriate therapeutic approach for patients is still an unmet goal in IgAN. Disease has a proven immunological origin, although multiple pathways remain unknown. Presumably, different antigens cause immune reactions that start on mucosal surfaces, continue in the systemic circulation and are finalized in the glomeruli with local activation of native cells, attraction and accumulation of inflammatory cells, stimulation of the inflammatory cascade including complement activation, all ending in renal scarring [1, 2]. None of the above-mentioned processes has yet been clearly described. Conservative treatment based on RAASi and SGLT2i has a role in reducing RAS activity, controls hypertension, reduces glomerular hyperfiltration and preserves endothelial function [14, 15]. Apparently, such treatment is mainly based on the evidence of laboratory beneficial effects and does not take into account disease's immunological profile. However, both treatment modalities efficiently reduce proteinuria levels, and therefore they should be administered in addition to specific treatment. Systemic steroids have been considered an effective therapeutic option, as they reduce inflammation and seem to prevent deterioration of kidney function [12, 13, 25]. However, their broad use is limited by the high rate of serious side effects [26, 27]. Finding a new and effective treatment with fewer side effects would be promising news for IgAN patients [28].

Despite our novel and important findings, there are indeed some limitations in our study, as this was not a randomized controlled trial, and did not run under austere and controlled conditions, participating patients were older than the general population in IgAN and covered a wide range of histological lesions in kidney biopsy, and a wide range of time since diagnosis.

## CONCLUSIONS

Herein, we presented the preliminary results of a multicenter study that describe the prompt proteinuria changes after commencement of TRB, focusing on the impact of related clinical and histological parameters.

Time elapsed since disease diagnosis, preserved renal function, severity of proteinuria and active, not chronic, lesions on kidney biopsy were found the most important parameters that may determine response to treatment, and their combination can demonstrate the profile of patients who will benefit from TRB treatment.

## Supplementary Material

sfae364_Supplemental_File

## Data Availability

The data underlying this article cannot be shared publicly due to the privacy of individuals who participated in the study. The data will be shared on reasonable request to the corresponding author.
