# Supplementary material for: Factors influencing early response of IgA nephropathy following targeted-release budesonide (TRB) treatment: preliminary results from a multicenter study
Source: Clin Kidney J. 2024 Nov 19;18(1):sfae364. doi: 10.1093/ckj/sfae364 (PMC11833314; doi:10.1093/ckj/sfae364)
Supplement: sfae364_Supplemental_File [file sfae364_Supplemental_File.docx]

**Supplementary Material**

**Suppl Table 1.** Each patient’s kidney biopsy is evaluated according to Oxford classification

| No | Oxford classification score | Mesangial hypercellularity (M0/M1) | Endocapillary hypercellularity (E0/E1) | Segmental glomerulosclerosis (S0/S1) | Tubular atrophy/interstitial fibrosis (T0/T1/T2) | Cellular/ fibro cellular crescents (C0/C1/C2) |
| --- | --- | --- | --- | --- | --- | --- |
| 1 | M1E1S1T1-C1 | M1 | E1 | S1 | T1 | C1 |
| 2 | M1E1S1T1-C0 | M1 | E1 | S1 | T1 | C0 |
| 3 | M0E1S0T0-C0 | M0 | E1 | S0 | T0 | C0 |
| 4 | M1E0S1T0-C0 | M1 | E0 | S1 | T0 | C0 |
| 5 | M1E1S1T1-C1 | M1 | E1 | S1 | T1 | C1 |
| 6 | M1E1S1T0-C0 | M1 | E1 | S1 | T0 | C0 |
| 7 | M1E0S1T1-C1 | M1 | E0 | S1 | T1 | C1 |
| 8 | M1E0S1T1-C0 | M1 | E0 | S1 | T1 | C0 |
| 9 | M1E1S1T1-C0 | M1 | E1 | S1 | T1 | C0 |
| 10 | M0E0S0T1-C0 | M0 | E0 | S0 | T1 | C0 |
| 11 | M1E0S0T1-C0 | M1 | E0 | S0 | T1 | C0 |
| 12 | M1E0S1T0-C0 | M1 | E0 | S1 | T0 | C0 |
| 13 | M1E0S1T1-C0 | M1 | E0 | S1 | T1 | C0 |
| 14 | M1E0S1T1-C0 | M1 | E0 | S1 | T1 | C0 |
| 15 | M1E0S1T1-C0 | M1 | E0 | S1 | T1 | C0 |
| 16 | M1E0S1T0-C0 | M1 | E0 | S1 | T0 | C0 |
| 17 | M1E0S1T1-C0 | M1 | E0 | S1 | T1 | C0 |
| 18 | M1E0S1T1-C0 | M1 | E0 | S1 | T1 | C0 |
| 19 | M1E1S1T1-C0 | M1 | E1 | S1 | T1 | C0 |
| 20 | M1E1S1T0-C0 | M1 | E1 | S1 | T0 | C0 |
| 21 | M1E1S1T0-C0 | M1 | E1 | S1 | T0 | C0 |
| 22 | M1E1S1T1-C0 | M1 | E1 | S1 | T1 | C0 |
| 23 | M1E1S1T1-C0 | M1 | E1 | S1 | T1 | C0 |
| 24 | M1E1S1T0-C0 | M1 | E1 | S1 | T0 | C0 |
| 25 | M1E1S1T0-C0 | M1 | E1 | S1 | T0 | C0 |
| 26 | M1E1S1T0-C0 | M1 | E1 | S1 | T0 | C0 |
| 27 | M1E1S1T1-C0 | M1 | E1 | S1 | T1 | C0 |
| 28 | M0E1S0T0-C1 | M0 | E1 | S0 | T0 | C1 |
| 29 | M1E0S1T1-C1 | M1 | E0 | S1 | T1 | C1 |
| 30 | M1E1S1T1-C1 | M1 | E1 | S1 | T1 | C1 |
| 31 | M1E1S1T1-C1 | M1 | E1 | S1 | T1 | C1 |
| 32 | M1E1S1T1-C1 | M1 | E1 | S1 | T1 | C1 |
| 33 | M1E1S1T0-C1 | M1 | E1 | S1 | T0 | C1 |
| 34 | M1E1S1T1-C1 | M1 | E1 | S1 | T1 | C1 |
| 35 | M1E1S1T1-C1 | M1 | E1 | S1 | T1 | C1 |
| 36 | M1E1S1T0-C1 | M1 | E1 | S1 | T0 | C1 |
| 37 | M1E1S1T1-C1 | M1 | E1 | S1 | T1 | C1 |
